# N1 lymph node detection in lymph node harvesting in non-small cell lung cancer: Formaldehyde exposure is a drawback?

**DOI:** 10.1186/s13019-023-02380-5

**Published:** 2023-10-10

**Authors:** Sevinc Citak, Talha Dogruyol, Serkan Bayram, Mustafa Vayvada, Serda Kanbur Metin, Volkan Baysungur

**Affiliations:** 1 Department of Thoracic Surgery, Kartal Kosuyolu High Specialization Education & Research Hospital, Istanbul, Turkey; 2 Department of Thoracic Surgery, Kartal Dr Lutfi Kirdar City Hospital, Istanbul, Turkey; 3 Department of Thoracic Surgery, Sureyyapasa Chest Diseases and Thoracic Surgery Training & Research Hospital, Istanbul, Turkey

**Keywords:** Non-small cell lung cancer, Lymph node detection, Survival rates

## Abstract

**Objective:**

This study aimed to evaluate the effect of lymph node dissection method on staging results, diagnosis of tumor metastasis in single or multiple N1 lymph nodes and survival rates in patients with non-small cell lung cancer (NSCLC).

**Methods:**

Patients with NSCLC who underwent anatomic resection between September 2014 and October 2019 were examined prospectively. All patients with similar clinico-demographic characteristics were randomly assigned to either the surgical group (n = 83) or the pathology group (n = 87). Lymph node dissection was performed by the surgeon in the surgical group and by the pathologists after formaldehyde exposure in the pathology group. Data were analyzed according to formaldehyde exposure, N1 positivity, and number of N1 positive lymph nodes.

**Results:**

There were no significant differences in N1 lymph node positivity between the two groups (p = 0.482). On average 9.08 lymph node sampling was performed in the surgical group and 2.39 in the pathology group (p = 0.0001). Multiple lymph node involvement was significantly higher in the surgical group (P = 0.0001) than in the pathology group.

**Conclusion:**

It is easier to detect lymph node involvement without introducing formaldehyde into the sample. We recommend that N1 lymph node dissection be performed on fresh specimens to detect more lymph node involvement.

## Introduction

Lung cancer is the most common cause of cancer-related deaths globally, both, in men and women. An estimated 2.09 million new cases are detected worldwide, accounting for 11.6% of all cancers [1]. Surgery remains the only potentially curative treatment for patients with early stage non-small cell lung cancer (NSCLC). Surgical excision of the tumor, along with regional and mediastinal lymph nodes, is essential for complete resection. The Tumor, Nodes, Metastasis (TNM) staging system is established as the most accurate prognostic tool for patients with NSCLC [2].

The current National Comprehensive Cancer Network (NCCN) guidelines recommend complete surgical resection followed by a platinum-based dual adjuvant chemotherapy regimen for patients with pathological N1 disease [3, 4]. Various randomized controlled trials and subsequent meta-analyses indicate a reduction in disease recurrence and an overall 5-year survival advantage among all patients treated as per the aforementioned modality [5–9]. Patients with N1 NSCLC represent a heterogeneous population, with varying long-term survival rates. The prognosis and recurrence pattern are thought to be especially affected by lymph node involvement [10]. Therefore, accurate staging of lymph node involvement is essential for determining the appropriate treatment modality for patients with NSCLC.

Despite advances in imaging technology, N1 lymph node metastasis is challenging to detect preoperatively and is often not fully diagnosed until the postoperative pathological examination. Patients with clinical N1 disease are considered surgical candidates, based on the current TNM staging system. However, upstaging or downstaging may occur in the nodal condition defined by the pathological examination after surgery [10, 11].

Mediastinal lymph node dissection should be performed even in patients with clinical stage I NSCLC. However, not all N1 lymph nodes are dissected during surgery. N1 lymph nodes can be dissected on the back table and then sent for pathological examination prior to coming in contact with the formaldehyde solution; this is done in order to accurately complete the staging procedure as formaldehyde is thought to cause deterioration in the specimen’s structure, including shrinkage and changes in parenchyma volume, thus creating false negatives. In our study, we aimed to examine the effects of the lymph node dissection method on staging outcomes, diagnosis of tumor metastasis in one or more N1 lymph nodes, and its impact on survival rates among patients with NSCLC who underwent surgical resection.

## Methods

Patients who underwent lobectomy, bilobectomy and pneumonectomy for NSCLC at our clinic between September 2014 and October 2019 were examined prospectively. All patients were preoperatively diagnosed with NSCLC and were assessed using positron emission tomography (PET/CT) for clinical staging before surgical treatment. The exclusion criteria were (a) N2 lymph node positivity in preoperative mediastinal staging, (b) N2 lymph node positivity after resection, (c) neoadjuvant radiotherapy and/or chemotherapy, (d) distant organ metastasis, (e) synchronous tumor, (f) sublobar resection, and (g) incomplete surgical resection of the tumor (R1 or R2). In this study, the primary endpoint is the effect of the harvesting method in detecting N1 lymph node involvement. The secondary endpoint is the effect of N0, N1 and multiple N1 lymph nodes on survival. The study protocol was approved by the Local Ethical Committee of Clinical Research. This study was conducted in accordance with the principles of the Declaration of Helsinki. Informed consent was obtained from all patients in the study.

### Patient groups

Patients were divided into two groups based on the N1 stations lymph node dissection technique. In the right lung dissected lymph node stations were 10 hilar, 11 interlobar (superior, inferior), 12 lobar (superior, inferior), 13 segmental (apical, posterior, anterior, medial, lateral, superior, laterobasal, mediobasal, anterobasal, posterobasal) and 14 subsegmental (apical, posterior, anterior, medial, lateral, superior, laterobasal, mediobasal, anterobasal, posterobasal). In the left lung; 10 hilar, 11 interlobar (superior,inferior), 12 lobar (superior, inferior), 13 segmental (apicoposterior, anterior, superior lingula, inferior lingula, superior, laterobasal, anteromediobasal, posterobasal), 14 subsegment(apicoposterior, anterior, superior lingula, inferior lingula, superior, laterobasal, anteromediobasal, posterobasal). Multiple N1 lymph node involvement was defined as pathological positivity in different N1 lymph node stations (stations 10-11-12-13-14). Patients with similar clinico-demographic characteristics including tumor size, and histology were randomly assigned to surgical and pathology groups. In the surgical group, N1 lymph node dissection was performed on the back table by the surgical team after resection. In the pathology group, the specimen was exposed to formaldehyde solution and transferred to the pathology laboratory; N1 lymph node dissections were then performed by pathologists in the pathology laboratory.

### Technique

In the surgical group, the N1 lymph nodes were dissected from the center to the periphery. The parenchyma was dissected over the most central bronchus (lobe bronchus for lobectomy, main bronchus for pneumonectomy) towards the periphery. Care was taken not to damage the integrity of the bronchial tree during dissection and to completely remove N1 lymph nodes. After surgical back table dissection, the removed lymph nodes were placed in formaldehyde and sent to the pathology laboratory.

N1 lymph node dissection was similarly performed in the pathology group by the pathologist. While the parenchyma was dissected towards the periphery over the most central bronchus, unlike in the surgical group, the bronchus was cut and the lymph nodes were dissected by moving distally over the bronchial tree. However, the specimens of the patients in this group were exposed to formaldehyde solution before dissection.

Patients’ age, sex, number of lymph node dissections, N1 positivity and the number of N1 positive lymph node stations, tumor dimensions, tumor status, and stages according to the 8th TNM system, histological types, tumor differentiation, visceral pleura involvement, type of resection, operation side, follow-up period, and survival were analyzed. In terms of N1 involvement, groups with no pathological N1 disease, single N1, or multiple N1 were examined for survival. Adjuvant chemotherapy was prescribed in our institution according to the oncology council decision. Adjuvant chemotherapy was recommended for patients with N1 disease and evaluated on a case-by-case basis in T3/4 patients. Cisplatin + vinorelbine combination was used as a standard regimen for patients requiring adjuvant chemotherapy in the absence of contraindications.

### Statistical analysis

Statistical analyses were performed using the Number Cruncher Statistical System (NCSS) 2017 Statistical Software (Utah, USA) package program. In addition to descriptive statistical methods (mean, standard deviation) in the evaluation of the data, the distribution of the variables was examined with the Shapiro–Wilk normality test, one-way analysis of variance, for the comparison of the normally distributed variable and independent t-test for the comparison of the paired groups. The Kruskal–Wallis test was used for intergroup comparison of non-normally distributed variables, Mann–Whitney U test was used for pairwise comparisons, and chi-square test was used for qualitative data comparisons. Kaplan–Meier analysis and log-rank test were used to determine the survival of the groups. The results were evaluated at a significance level of p < 0.05.

### Limitations

This study was limited by its small sample size, as it was a single-center study. The insufficient number of patients included in the study may have caused certain differences in the data.

## Results

**Fig. 1:**
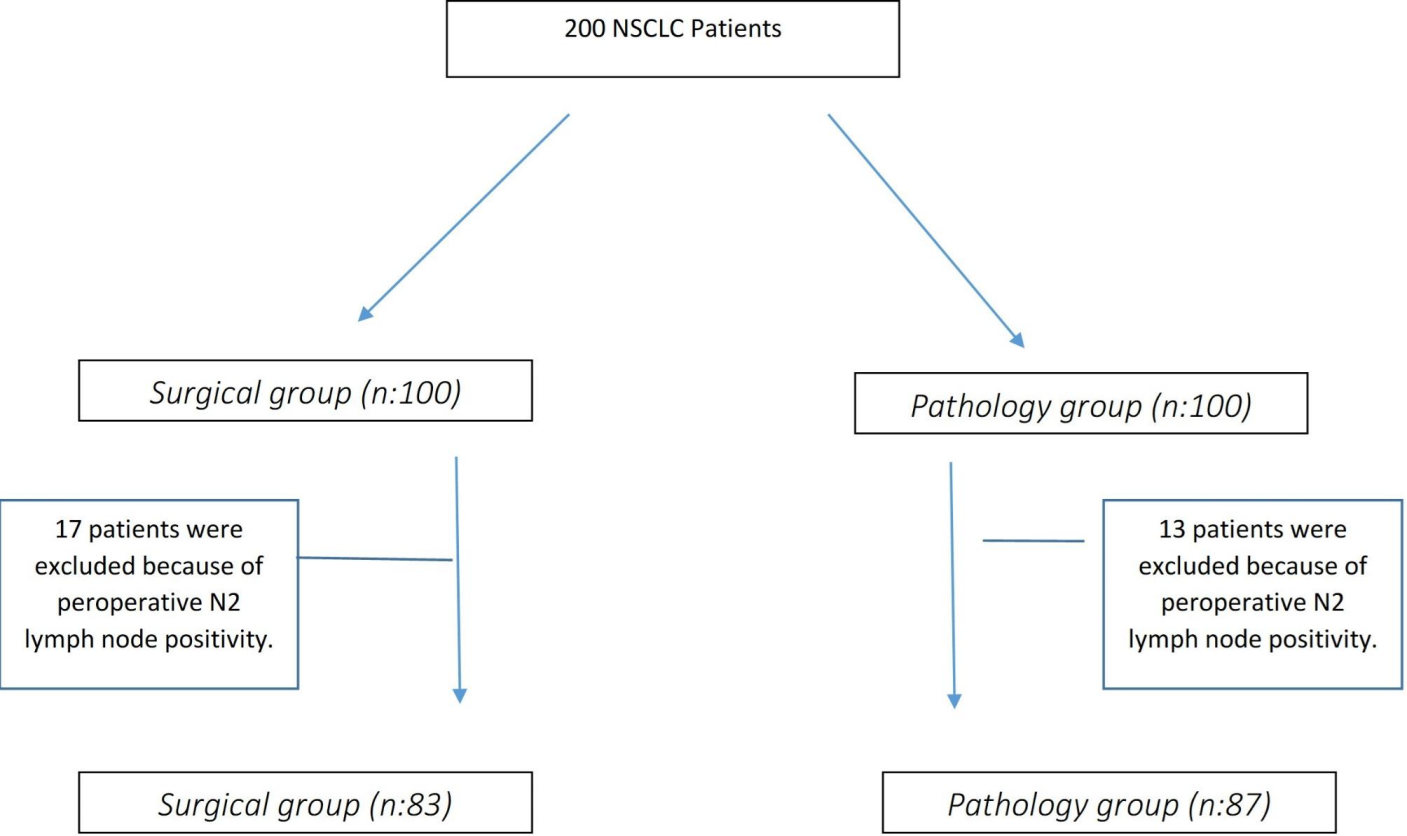
Flow diagram of registered patients

After applying the exclusion criteria, 170 patients were enrolled in the study, with 83 patients in the surgical group and 87 patients in the pathology group (Fig. 1). Of the total participants, 90.8% in the surgical group and 89.1% in the pathology group were male. There was no significant difference between the mean age and sex in both the groups (p = 0.587 and p = 0.720, respectively). In both groups, 44 patients underwent right-sided surgery. Left-sided surgery was performed on 43 and 39 patients in the surgical and pathology groups, respectively. The most common resection type was right upper lobectomy in both the groups. No significant difference was observed in terms of the operation side and type of resection distribution between the pathology and surgical groups (p = 0.751). No significant differences were observed between histological subtypes and tumor differentiation distributions (p = 0.980 and p = 0.848, respectively). However, the incidence of visceral pleural involvement was significantly higher in the surgical group than in the pathology group (p = 0.022) (Table 1).

**Table 1:** Demographic and clinical features of pathology and surgical groups

		Pathology Group	Surgical Group	p
**Age (** ***years*** **)**		61,8 ± 7,2	61,1 ± 9	0,587
**Sex**	Male	79 (90,8)	74 (89,1)	0,720
	Female	8 (9,2)	9 (10,8)	
**Side**	Right	44 (50,5)	44 (53)	0,751
	Left	43 (49,4)	39 (46,9)	
**Resection Type**	LLL	16 (18,3)	10 (12)	0,349
	LP	12 (13,7)	11 (13,2)	0,918
	LUL	15 (17,2)	18 (21,6)	0,590
	RBI	3 (3,4)	4 (4,8)	0,949
	RBS	2 (2,3)	1 (1,2)	0,588
	RLL	10 (11,4)	7 (8,4)	0,682
	RML	4 (4,6)	7 (8,4)	0,481
	RP	4 (4,6)	3 (3,6)	0,747
	RUL	21 (24,1)	22 (26,5)	0,858
**Histology**	Adenocarcinoma	40 (45,9)	38 (45,7)	0,980
	Squamous cell carcinoma	47 (54,0)	45 (54,2)	
**Tumor Differentiation**	Well	10 (11,4)	8 (9,6)	0,848
	İntermadiate	52 (59,7)	53 (63,8)	
	Poor	25 (28,7)	22 (26,5)	
**Visceral pleural involvement status**	No	76 (87,3)	61 (73,4)	0,022
	Yes	11 (12,6)	22 (26,5)	
**T Pathology Size (** ***mm*** **)**		31,7 ± 12,96	31,24 ± 14,95	0,830
**T Phase (according to TNM 8)**	1 A	0 (0,0)	3 (3,6)	0,114
	1B	14 (16,0)	16 (19,2)	0,688
	1 C	22 (25,2)	22 (26,5)	0,995
	2 A	34 (39,0)	24 (28,9)	0,845
	2B	11 (12,6)	8 (9,6)	0,705
	3	6 (6,9)	10 (12,0)	0,375
**Number of N1 lymph node dissections performed**		2,39 ± 2,58	9,08 ± 4,14	0,0001
**N1 lymph node status**	No	64 (73,5)	57 (68,6)	0,482
	Yes	23 (26,4)	26 (31,3)	
**Number of positive N1 lymph node involvement**	None	64 (73,5)	57 (68,6)	0,0001
	Single	23 (26,4)	8 (9,6)	
	Multiple	0 (0,0)	18 (21,6)	
**TNM 8 Staging**	1A1	0 (0,0)	3 (3,6)	0,227
	1A2	14 (16,0)	15 (18,0)	0,889
	1A3	22 (25,2)	18 (21,6)	0,709
	1B	16 (18,3)	15 (18,0)	0,978
	2 A	8 (9,2)	3 (3,6)	0,243
	2B	25 (28,7)	20 (24,1)	0,609
	3 A	2 (2,3)	9 (10,8)	0,055
**Follow-up time (** ***month*** **)**		27,9 ± 18,36	28,9 ± 17,11	0,701

Value are expressed as mean (min.-max. range) or N (%); LLL, left lower lobectomy; LP, left pneumonectomy; LUL, left upper lobectomy; RBI, right bilobectomy inferior; RBS, right bilobectomy superior; RLL, right lower lobectomy; RML, right middle lobectomy; RP, right pneumonectomy; RUL, right upper lobectomy; TNM tumor lymph node metastasis

There was no significant difference in tumor size and T stage as per the 8th TNM staging between the pathology and surgical groups (p = 0.850). No significant difference was observed in N1 lymph node positivity between the two groups either (p = 0.482). However, the number of dissected lymph nodes and the number of positive multiple lymph nodes were significantly higher in the surgical group than in the pathology group (p = 0.0001). On average 9.08 lymph node sampling was performed in the surgical group (2.39 in the pathology group (p = 0.0001)). Lymph node positivity was not detected in 64 patients (73.5%) in the pathology group, while single N1 positivity was detected in 23 patients (26.4%); multiple N1 positivity was not detected. In the surgical group, N1 positivity was not detected in 57 (68.7%) cases whereas single N1 positivity was observed in 8 patients (9.6%); multiple N1 positivity was observed in 18 (21.6%) cases. No significant difference was observed between the pathology and surgical groups in terms of N0 and N1 statuses (p = 0.482) (Table 1).

In the N0 group, 59 patients underwent surgery for adenocarcinoma and 62 patients had squamous cell carcinoma. In the single N1 group, 16 patients had adenocarcinoma and 15 patients had squamous cell carcinoma. In the multiple N1 group, three patients underwent surgery for adenocarcinoma and 15 patients for squamous cell carcinoma. When the groups were compared, a significant difference was observed between the histological distributions of the N0, single N1, and multiple N1 groups (p = 0.03). The incidence of squamous cell carcinoma was found to be significantly higher in the multiple N1 group (p = 0.03) (Table 2).

**Table 2:** Demographic and clinical features of patients acording to lymph node involvement

			No Lymph Node Involvement		Single Lymph Node Involvement		Multiple Lymph Node Involvement		p
**Age (** ***years*** **)**			61,4 ± 8		62 ± 8		61,2 ± 9,2		0,933
**Sex**		Male	107	(88,4)	28	(90,3)	18	(100)	0,311
		Female	14	(11,5)	83	(9,6)	0	(0,0)	
**Pathology Size (** ***mm*** **)**			29,2 ± 12,5		34,2 ± 15,1		42,0 ± 15,6		0,0001
**T Phase (according to TNM 8)**		1 A	3	(2,4)	0	(0,0)	0	(0,0)	0,539
		1B	29	(23,9)	0	(0,0)	1	(5,5)	0,003
		1 C	40	(33)	1	(3,2)	3	(16,6)	0,002
		2 A	31	(25,6)	21	(67,7)	6	(33,3)	0,0001
		2B	11	(9,1	6	(19,3)	2	(11,1)	0,270
		3	7	(5,7)	3	(9,6)	6	(33,3)	0,009
**TNM 8 stage**		1A1	3	(2,4)	0	(0)	0	(0)	0,539
		1A2	29	(23,9)	0	(0)	0	(0)	0,008
		1A3	40	(33,0)	0	(0)	0	(0)	0,0001
		1B	31	(25,6)	0	(0)	0	(0)	0,005
		2 A	11	(9)	0	(0)	0	(0)	0,092
		2B	5	(4,1)	28	(90,3)	12	(66,6)	0,0001
		3 A	2	(1,6)	3	(9,6)	6	(33,3)	0,0001
**Histology**		Adenocarcinoma	59	(48,7)	16	(51,6)	3	(16,6)	0,03
		Squamous cell carcinoma	62	(51,2)	15	(48,3)	15	(83,3)	
**Tumor Differentiation**		Well	15	(12,4)	2	(6,4)	1	(5,5)	0,408
		İntermadiate	76	(62,8)	20	(64,5)	9	(50)	
		Poor	30	(24,7)	9	(29)	8	(44,4)	
**Visceral pleural involvement status**		Yes	101	(83,4)	23	(74,2)	13	(72,2)	0,323
		No	20	(16,5)	8	(25,8)	5	(27,7)	
**Resection type**		LLL	21	(17,3)	3	(9,6)	2	(11,1)	0,498
		LPN	11	(9,0)	7	(22,5)	5	(27,7)	0,056
		LUL	25	(20,6)	5	(16,1)	3	(16,6)	0,810
		BLI	5	(4,1)	1	(3,2)	1	(5,5)	0,924
		BLS	2	(1,6)	1	(3,2)	0	(0)	0,699
		RLL	16	(13,2)	0	(0)	1	(5,5)	0,073
		RML	6	(4,9)	4	(12,9)	1	(5,5)	0,273
		RPN	3	(2,4)	2	(6,4)	2	(11,1)	0,175
		RUL	32	(26,4)	8	(25,8)	3	(16,6)	0,671
**Side**		Right	64	(52,8)	16	(51,6)	8	(44,4)	0,799
		Left	57	(47,1)	15	(48,3)	10	(55,5)	
**Follow-up (month)**			28,9 ± 17,77		21,9 ± 16,52		36,2 ± 16,3		0,031

**Fig. 2:**
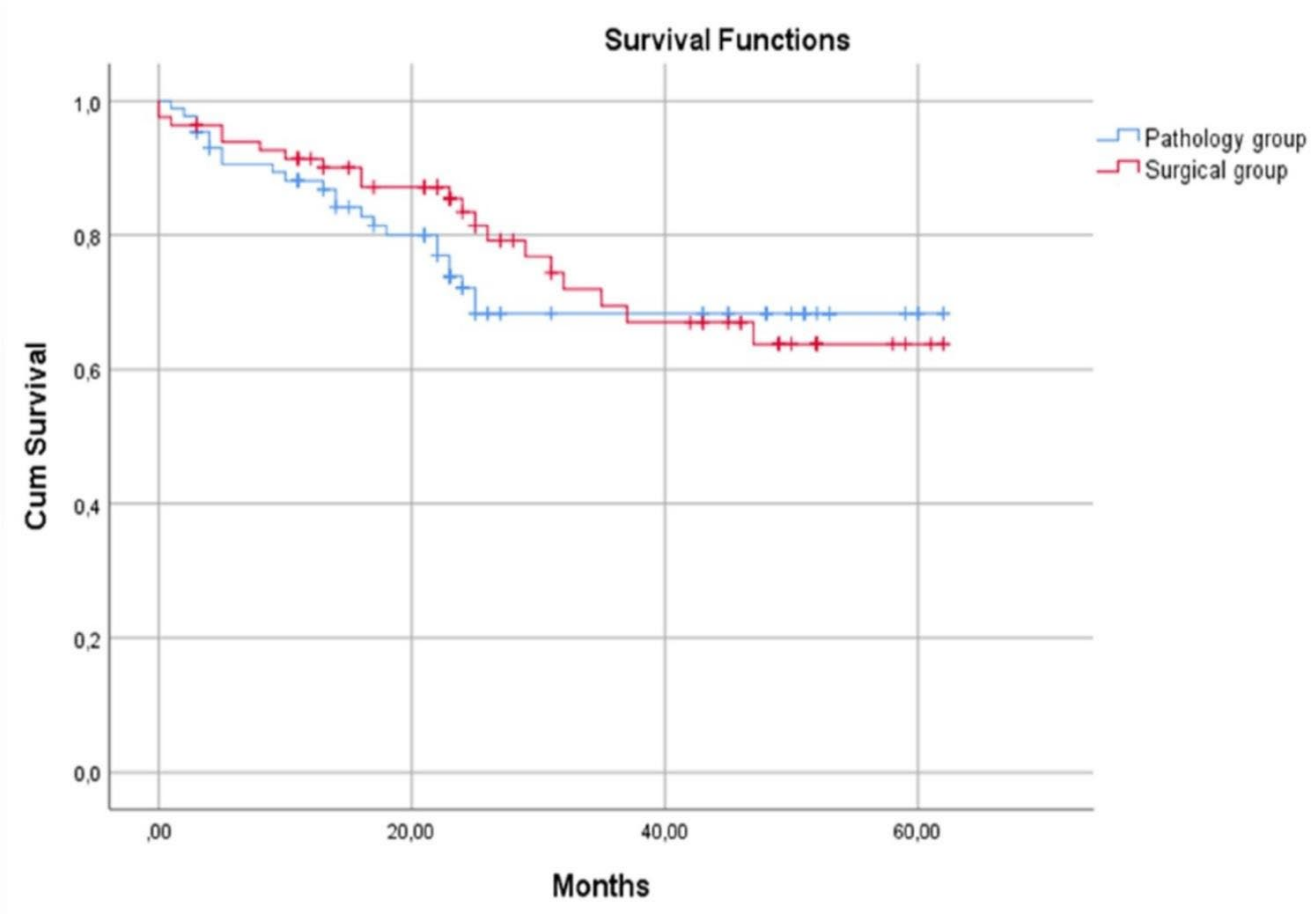
Survival of the pathology and surgery groups

Value are expressed as mean (min.-max. range) or N (%), TNM, tumor lymph node metastasis; LLL, left lower lobectomy; LPN, left pneumonectomy; LUL, left upper lobectomy; RBI, right bilobectomy inferior; RBS, right bilobectomy superior; RLL, right lower lobectomy; RML, right middle lobectomy; RP, right pneumonectomy; RUL, right upper lobectomy;

In the pathology group, the 1-year survival rate was 91.4%, 3-year survival rate was 69.4%, and 5-year survival rate was 63.8%. For the surgical group, the 1-year survival rate was 88.1%, 3-year survival rate was 68.3%, and 5-year survival rate was 68.3%. There was no significant difference in the survival rates between the groups regardless N0 land N1 diseases. (p = 0.198) (Fig. 2).

**Fig. 3:**
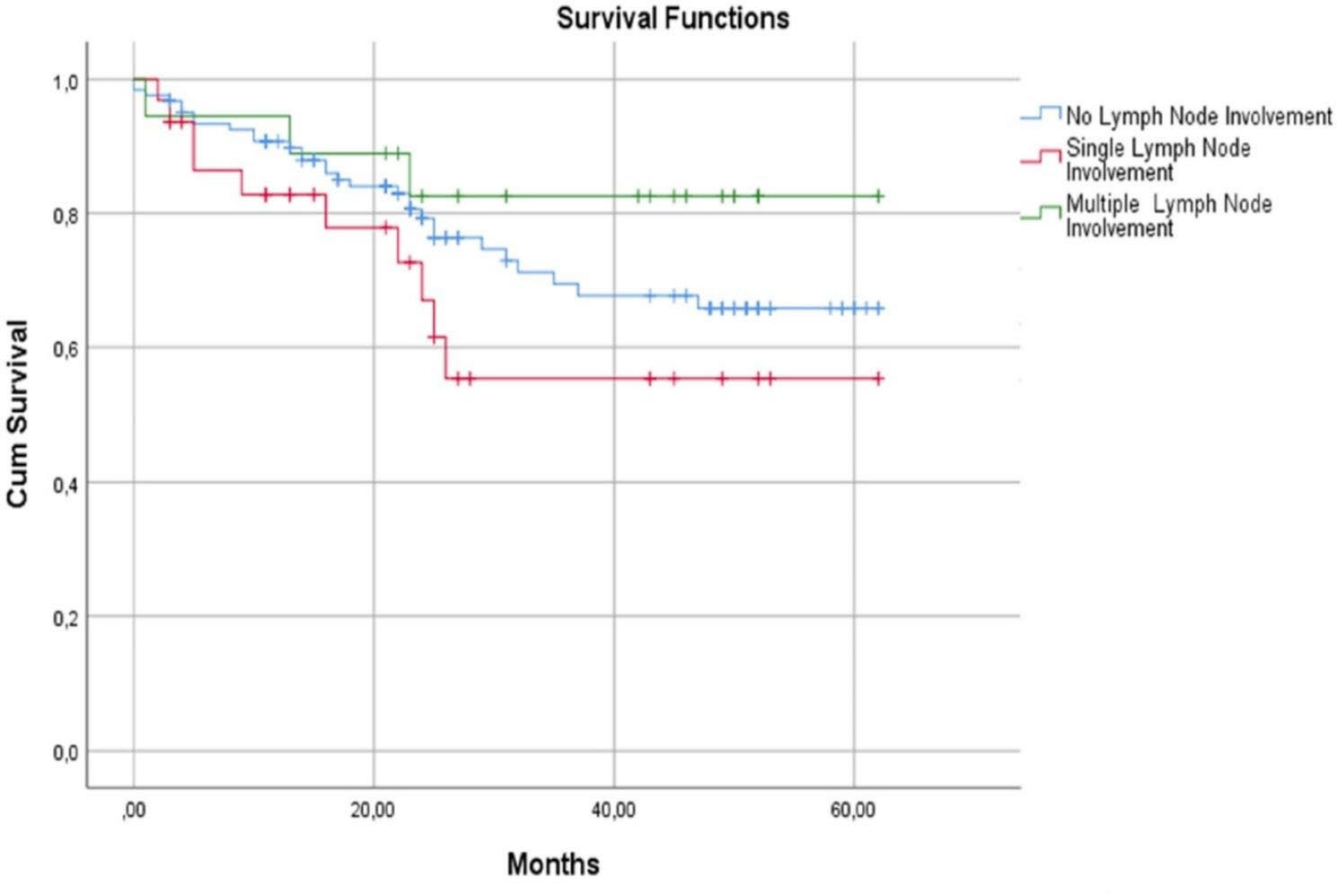
Kaplan-Meier survival curves for lymph node involvement

In the group without lymph node involvement, the 1-year survival rate was 95%, 3-year survival rate was 69.5%, and 5-year survival rate was 65.9% (median 47.66 ± 2.19 months). The 1-year survival rate of the group with single lymph node involvement was 82.8%, 3-year survival was 55.4%, and 5-year survival was 55.4% (median 36.06 ± 4.02 months). The 1-year survival in the group with multiple lymph node involvement was 94.4%, 3-year survival was 88.9%, and 5-year survival was 82.5% (median 50.71 ± 4.31 months). No significant difference was observed between the survival rates of the groups (p = 0.635) (Fig. 3).

## Discussion

In our study, it was found that dissecting the surgical specimen before formaldehyde exposure provided more accurate staging since the dissected number of N1 lymph nodes and multiple N1 positivity was higher in the surgical group than in the pathology group. Lymph node involvement is one of the most critical factors determining the prognosis of NSCLC. NSCLC cases with mediastinal lymph node involvement have a poor prognosis. Therefore, intraoperative mediastinal lymph node sampling/dissection is important for staging and deciding the course of the treatment. The T factor was revised in the last TNM stage, since one of the reasons for the differences in survival was the size of the mass. While it is planned to provide more accurate predictions with the changes in the T factor in the 8th staging, the debates on the detection of N1 lymph node involvement and prognosis continue. Variable results in patient survival suggest that different prognostic factors exist; studying these variables is imperative to provide the ideal treatment modality to the patient for better prognosis.

Maeshima et al. emphasized that multiple N1 positivity was a poor prognostic factor [12]. The effect of median survival on lymph node positivity was observed, and it was pointed out that survival decreased as the rate of positive lymph nodes increased. The number of lymph node involvement has been reported as an independent prognostic factor [13]. It is thought that it may be the source of the severe difference in survival in patients without N2. Lymph node dissection allows evaluation of the rate of positive lymph nodes in N1 disease. In our study, we examined lymph node dissections performed by the surgical team on the surgical table prior to formaldehyde exposure and those performed by pathologists after formaldehyde exposure. We found that multiple N1 lymph node positivity was higher in the surgical group. Formaldehyde exposure affects the structure of the lobes and reduces their volume. Lymph node dissection becomes difficult in tissue that loses tissue vitality with formaldehyde exposure.

In a series of 1742 patients with NSCLC, hilar lymph nodes were divided into three groups according to N1 lymph node involvement: single hilar, single interlobar and multiple N1 positive. These groups were compared among themselves as well as with patients with single N2. It was observed that patients with multiple N1 and patients with single N2 had similar survival rates [14]. Wang et al. stated that the number of positive lymph nodes was not associated with long-term survival although location-based classification and positive lymph nodes were important in prognosis in a meta-analysis on the prognostic importance of the N1 classification [15]. In our study, no significant difference was observed between the N1 lymph node positivity determination of the pathology and surgical groups. However, the number of lymph nodes dissected in the surgical group was significantly higher than that in the pathology group. Therefore, we found more multiple N1 positivity in the surgical group. Although survival was expected to be worse in the multiple N1 group, no significant difference was observed between the survival rates of the N0, single N1, and multiple N1 groups in the surgical and pathology groups. These results suggest that some multiple N1 patients in the pathology group may not have been detected in our study. This similarity may be due to the development of postoperative oncologic treatment modality and increased access to a multidisciplinary team. We believe that further studies are needed to investigate how multiple N1 lymph node positivity affects survival rates and whether or not it influences the prognosis of the case.

It is generally pointed out that N1 lymph node involvement is more common in patients with adenocarcinoma than in those with squamous cell carcinoma [16, 17]. No differences were observed between the histological subtypes in terms of lymph node metastasis in our study. In addition, squamous cell carcinoma was observed more frequently in patients with multiple N1 tumors. The possibility of lymph node involvement was high in patients with N1 positivity and tumor diameters > 10 mm [18–20]. Studies with larger patient groups need to be conducted to confirm the lymph node correlation between T stage and TNM.

## Conclusion

A multidisciplinary approach is required to treat patients with NSCLC. Surgical resection is considered the most important curative option. Survival differences in patients undergoing surgery have shown the importance of lymph node involvement in prognosis, and these differences have led to increasingly detailed staging of patients. In this context, changes were made to the T and M groups during the eighth stage. The introduction of criteria such as more than one N1 lymph node involvement may increase the prognostic importance of staging and help determine better treatment strategies.

Formaldehyde complicates the dissection of hilar and peripheral lymph nodes, and specimens should be dissected without exposure to formaldehyde solution. Although not included in the 8th TNM staging, it is much easier to detect multiple N1 lymph node positivity of fresh specimens; it can be important for prognosis. We recommend performing N1 lymph node dissection on fresh specimens after the surgical resection of NSCLC for more accurate staging outcomes and determination of an accurate prognosis and treatment strategies.

Although the significant difference in the number of lymph nodes in the surgery and pathology groups suggests that there will be a difference in the pathology result, the result is not significant. This result shows that pathological dissections are also sufficient and the surgeon-pathologist harmony is excellent.

## Data Availability

All data generated or analyzed during this study are included in this published article.
